# Methionine Biosynthesis in *Staphylococcus aureus* Is Tightly Controlled by a Hierarchical Network Involving an Initiator tRNA-Specific T-box Riboswitch

**DOI:** 10.1371/journal.ppat.1003606

**Published:** 2013-09-12

**Authors:** Sonja M. K. Schoenfelder, Gabriella Marincola, Tobias Geiger, Christiane Goerke, Christiane Wolz, Wilma Ziebuhr

**Affiliations:** 1 Universität Würzburg, Institut für Molekulare Infektionsbiologie, Würzburg, Germany; 2 Queen's University Belfast, Centre for Infection and Immunity, Belfast, United Kingdom; 3 Universität Tübingen, Interfakultäres Institut für Mikrobiologie & Infektionsmedizin, Tübingen, Germany; Vanderbilt University, United States of America

## Abstract

In line with the key role of methionine in protein biosynthesis initiation and many cellular processes most microorganisms have evolved mechanisms to synthesize methionine *de novo*. Here we demonstrate that, in the bacterial pathogen *Staphylococcus aureus*, a rare combination of stringent response-controlled CodY activity, T-box riboswitch and mRNA decay mechanisms regulate the synthesis and stability of methionine biosynthesis *metICFE-mdh* mRNA. In contrast to other *Bacillales* which employ S-box riboswitches to control methionine biosynthesis, the *S. aureus metICFE-mdh* mRNA is preceded by a 5′-untranslated *met* leader RNA harboring a T-box riboswitch. Interestingly, this T-box riboswitch is revealed to specifically interact with uncharged initiator formylmethionyl-tRNA (tRNA_i_^fMet^) while binding of elongator tRNA^Met^ proved to be weak, suggesting a putative additional function of the system in translation initiation control. *met* leader RNA/*metICFE-mdh* operon expression is under the control of the repressor CodY which binds upstream of the *met* leader RNA promoter. As part of the metabolic emergency circuit of the stringent response, methionine depletion activates RelA-dependent (p)ppGpp alarmone synthesis, releasing CodY from its binding site and thereby activating the *met* leader promoter. Our data further suggest that subsequent steps in *metICFE-mdh* transcription are tightly controlled by the 5′ *met* leader-associated T-box riboswitch which mediates premature transcription termination when methionine is present. If methionine supply is limited, and hence tRNA_i_^fMet^ becomes uncharged, full-length *met* leader/*metICFE-mdh* mRNA is transcribed which is rapidly degraded by nucleases involving RNase J2. Together, the data demonstrate that staphylococci have evolved special mechanisms to prevent the accumulation of excess methionine. We hypothesize that this strict control might reflect the limited metabolic capacities of staphylococci to reuse methionine as, other than *Bacillus*, staphylococci lack both the methionine salvage and polyamine synthesis pathways. Thus, methionine metabolism might represent a metabolic Achilles' heel making the pathway an interesting target for future anti-staphylococcal drug development.

## Author Summary

Prokaryote metabolism is key for our understanding of bacterial virulence and pathogenesis and it is also an area with huge opportunity to identify novel targets for antibiotic drugs. Here, we have addressed the so far poorly characterized regulation of methionine biosynthesis in *S. aureus*. We demonstrate that methionine biosynthesis control in staphylococci significantly differs from that predicted for other *Bacillales*. Notably, involvement of a T-box instead of an S-box riboswitch separates staphylococci from other bacteria in the order. We provide, for the first time, direct experimental proof for an interaction of a methionyl-tRNA-specific T-box with its cognate tRNA, and the identification of initiator tRNA_i_^fMet^ as the specific binding partner is an unexpected finding whose exact function in *Staphylococcus* metabolism remains to be established. The data further suggest that in staphylococci a range of regulatory elements are integrated to form a hierarchical network that elegantly limits costly (excess) methionine biosynthesis and, at the same time, reliably ensures production of the amino acid in a highly selective manner. Our findings open a perspective to exploit methionine biosynthesis and especially its T-box-mediated control as putative target(s) for the development of future anti-staphylococcal therapeutics.

## Introduction

Staphylococci are important skin and mucosa commensals but also major human pathogens. The most pathogenic species *Staphylococcus aureus* causes a wide range of diseases and, together with coagulase-negative staphylococci (CoNS), accounts for approximately 30 per cent of all hospital-acquired infections [1]. The development of antibiotic resistance in staphylococci increasingly limits therapeutic options and is a matter of major concern [2]. In recent years, studies into staphylococcal metabolism and its possible links to bacterial virulence have become a major focus of research but basic metabolic pathways remained largely unexploited in the development of new antibiotic drugs [3]. In this study, we investigate the regulation of methionine biosynthesis in staphylococci. Methionine and its chemical derivatives have important functions in the cell. For example, (formyl-)methionine is the universal N-terminal amino acid of nearly all proteins and therefore plays an eminent role in the initiation of protein biosynthesis. Moreover, the methionine derivative S-adenosylmethionine (SAM) serves as a methyl group donor in a variety of cellular processes and is the precursor molecule in polyamine synthesis [4]. Many microorganisms are able to synthesize methionine *de novo* and staphylococci employ the trans-sulfuration pathway to generate methionine [5]. Most bacteria from the order *Bacillales* are thought to control this pathway by SAM-binding S-box riboswitches [5], [6], [7]. Interestingly, *in silico* analysis predicts the presence of a T-box riboswitch in the 5′-untranslated region of the methionine biosynthesis operon (*metICFE-mdh* operon) in staphylococci [5], [6], [8], suggesting the use of alternative mechanisms to regulate methionine synthesis. T-box riboswitches are transcriptional control systems which have been extensively studied in *Bacillus subtilis* and other Firmicutes (reviewed in [6]). Their function is controlled by specific interactions and differential binding to charged and uncharged cognate tRNA, respectively, thus providing a means to “sense” the amino acid concentration in the cell [9]. T-box leader RNA/tRNA interaction essentially occurs at two sites: (i) the tRNA anticodon basepairs with the specifier-loop domain of the T-box leader RNA ensuring specific binding of the respective T-box element with its cognate tRNA; (ii) the free 3′-CCA end of an uncharged tRNA binds to the T-box motif, thereby triggering the formation and stabilization of an antiterminator which enables transcription of downstream genes [9]. In this study, we characterized interactions of the *metICFE-mdh* leader RNA with methionyl-tRNAs and demonstrate that they represent a functional T-box riboswitch that preferentially binds to initiator formylmethionyl-tRNA (tRNA_i_^fMet^). We further show that, in staphylococci, T-box control of methionine biosynthesis has a key role in a complex regulatory network that also involves stringent response-mediated CodY regulation and RNA decay to tightly control this pathway.

## Results

### A 5′ *met* leader RNA harboring a T-box riboswitch precedes the methionine biosynthesis operon

**Figure 1:**
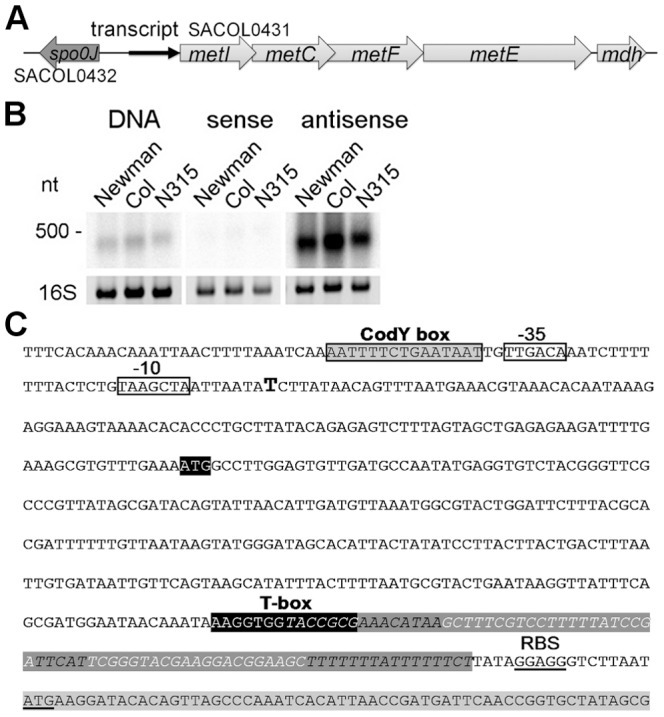
Determination of a leader RNA transcript upstream of methionine biosynthesis genes. (A) Overview of the genomic organization of the methionine biosynthesis operon. A transcript (black arrow) was detected upstream of *metI*. (B) Northern blot analysis of total RNA from three *S. aureus* strains (Newmann, COL and N315) for the intergenic region (IGR) between *spo0J* and *metI*. Radiolabeled probes were either the PCR product (DNA) or *in vitro* transcribed RNA of each strand (sense and antisense) of the IGR. The experiment was done in duplicate. Fragment sizes correspond to a high-range RNA ladder (Fermentas). The 16S rRNA is shown as a loading control in the corresponding agarose gel. (C) Sequence of the IGR upstream of *metI* in *S. aureus* COL. Transcription start of the *met* leader RNA, as experimentally determined by 5′ RACE, is indicated by a bold ‘T’; putative -35 and -10 promoter sites are boxed; the CodY binding motif is boxed in grey. The potential specifier box and the highly conserved T-box motif are shown in black; overlapping with the T-box is a predicted Rho-independent transcription terminator (in italics and dark grey). The Shine-Dalgarno sequence (SD) and the start codon of the *metI* gene (light grey) are underlined.

The methionine biosynthesis genes (*metI, metC, metF, metE, mdh* (metal-dependent hydrolase)) are organized in an operon-like structure and are annotated as SACOL0431 - SACOL0427 in *S. aureus* COL and as NWMN_0351 - NWM_0347 in *S. aureus* Newman, respectively, with *metF* being named *metH* in the latter strain (Figure 1A). Northern blot analysis using a double-stranded DNA probe confirmed the expression of a stable transcript of approximately 400 nucleotides (nt) from the intergenic region (IGR) upstream of *metI* (Figure 1B, left panel). Hybridization employing *in vitro*-transcribed RNA probes revealed that the orientation of the transcript was identical to that of the *metICFE-mdh* operon (Figure 1B, middle and right panels). 5′- and 3′-RACE experiments identified a single transcription start site and a transcript length of 439 nt (Figure 1C). Sequence analysis of various *S. aureus* and *S. epidermidis* strains demonstrated that the region is highly conserved (SI Figure S1) but lacks ribosomal binding sites and open reading frames, suggesting specific (non-coding) functions of the transcript, for example, as a 5′-untranslated region (5′-UTR) of the *metICFE-mdh* RNA. Interestingly, a putative binding site for the repressor protein CodY [10], [11] could be identified next to the 5′-UTR promoter region (Figure 1C). Most striking was the presence of a highly conserved canonical T-box sequence motif (5′-AAGGUGGUACCGCG-3′) which partially overlapped with a strong Rho-independent transcription termination signal in the 3′-portion of the transcript (Figure 1C). Overall, the transcript, which was named *met* leader RNA, harbored all the characteristics of previously characterized T-box riboswitches from *Bacillus subtilis*, and sequence alignments with *Bacillus* T-box systems led to a putative structural model of the *Staphylococcus met* leader RNA (SI Figure S2).

### The *met* leader RNA specifically binds uncharged tRNA_i_^fMet^

**Figure 2:**
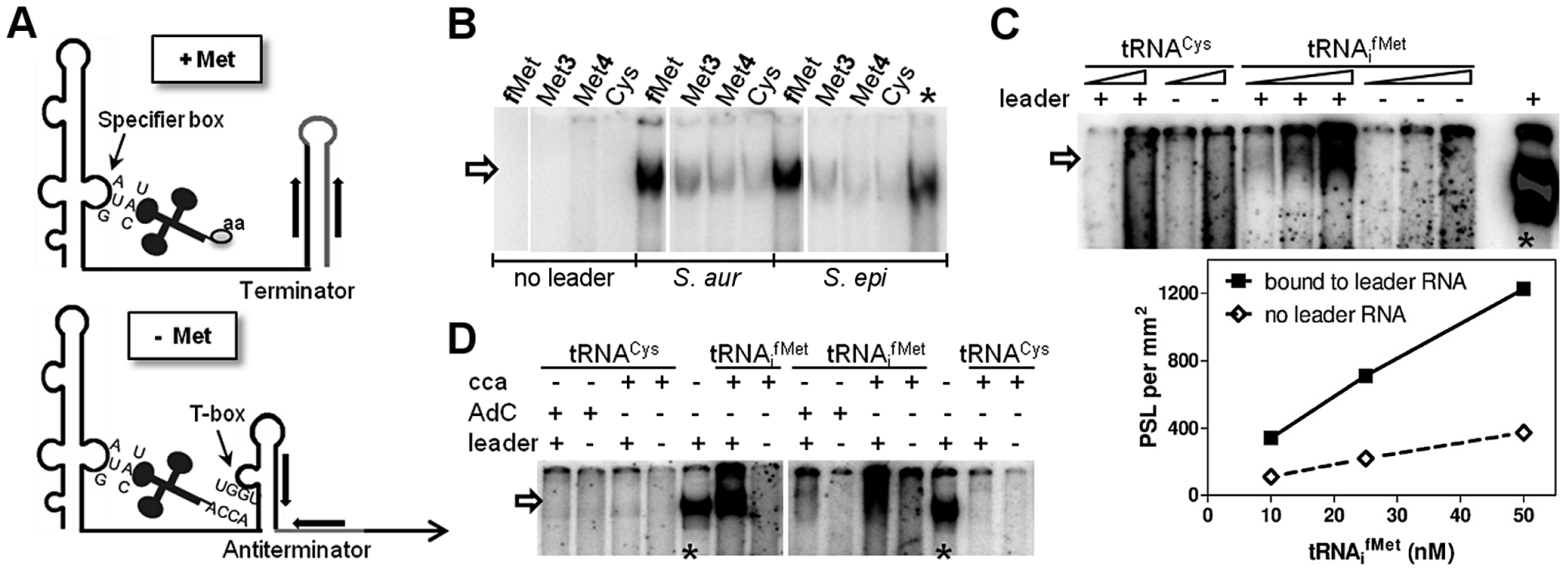
*In vitro* binding of *met* leader RNA to tRNAs. (A) Schematic of the binding interaction between tRNA and T-box leader RNA according to *Bacillus* T-box systems [9]. On the top (‘+ Met’), system under high intracellular levels of methionine, on the bottom (‘− Met’) under methionine starvation. Indicated are tRNAs with an amino acid (‘aa’, top) or a free 3′-CCA end (bottom) and the respective pairing sequences within the T-box leader RNA. (B–D) The *met* leader RNA was transcribed *in vitro* by T7 RNA polymerase from a defined DNA template. Where indicated, preformed and radiolabeled tRNA was present during the *in vitro* transcription (IVT) reaction. Samples were analyzed by a non-denaturing PAGE. Asterisks mark IVT *met* leader RNA reactions without tRNA, but with [α^32^P]-CTP present as an IVT efficiency control. The arrow indicates tRNAs bound to the *met* leader RNA. All experiments were performed at least twice. (B) Methionine-specific tRNA from the different genomic loci (tRNA_i_^fMet^, tRNA^Met3^ and tRNA^Met4^) or tRNA^Cys^ were present in the IVT. The leader RNA was transcribed from either the *S. aureus* COL or *S. epidermidis* RP62A sequence. (C) Increasing concentrations of each tRNA with 3′-CCA end (10 and 50 nM for tRNA^Cys^ and 10, 25 and 50 nM for tRNA_i_^fMet^, respectively) were present during IVT. Bound tRNA_i_^fMet^ was quantified by measuring the Photo-Stimulated Luminescence (PSL), which is proportional to the amount of radiation exposed to the IP plate. The PSL values are expressed per mm^2^ (y-axis) against the tRNA molarity (x-axis). (D) Either formylmethionine- (tRNA_i_^fMet^) or cysteine- (tRNA^Cys^) specific tRNA was present during IVT. Two different tRNA species were used: with a free 3′-CCA end (‘cca’) or with an additional cytosine (‘AdC’) at the 3′-CCA end to mimic amino acid charging [31].

First, we tested if the *Staphylococcus met* leader RNA interacts specifically with methionyl-tRNAs (Figure 2A). *met* leader RNA was *in vitro* transcribed in the presence of the appropriate radioactively labeled tRNA species. Binding between radioactively labeled tRNA and *in vitro*-transcribed *met* leader RNA was determined by non-denaturing polyacrylamide gel electrophoresis and autoradiography. Staphylococci genomes harbor four methionyl-tRNA gene loci, two of which (*tRNA-Met-1* and *-2*) being identical and representing the initiator tRNA_i_^fMet^. Binding studies using tRNA_i_^fMet^, tRNA^Met3^ and tRNA^Met4^, respectively, with free 3′-CCA ends revealed that the *met* leader RNA interacted strongly with tRNA_i_^fMet^ while interactions with tRNA^Met3^ and tRNA^Met4^ proved to be weak (Figure 2B). tRNA_i_^fMet^ binding to *met* leader RNA increased linearly within a 5-fold molar range (Figure 2C). In contrast, binding was abolished when the 3′-end of tRNA_i_^fMet^ included one additional cytosine, mimicking a charged tRNA molecule (3′-CCAC; AdC in Figure 2D). Also, no interaction was detectable in the presence of cysteinyl-tRNA, regardless of whether 3′-CCA or 3′-CCAC was present at the 3′ end (Figure 2C, D).

### tRNA_i_^fMet^ binding requires the T-box motif

**Figure 3:**
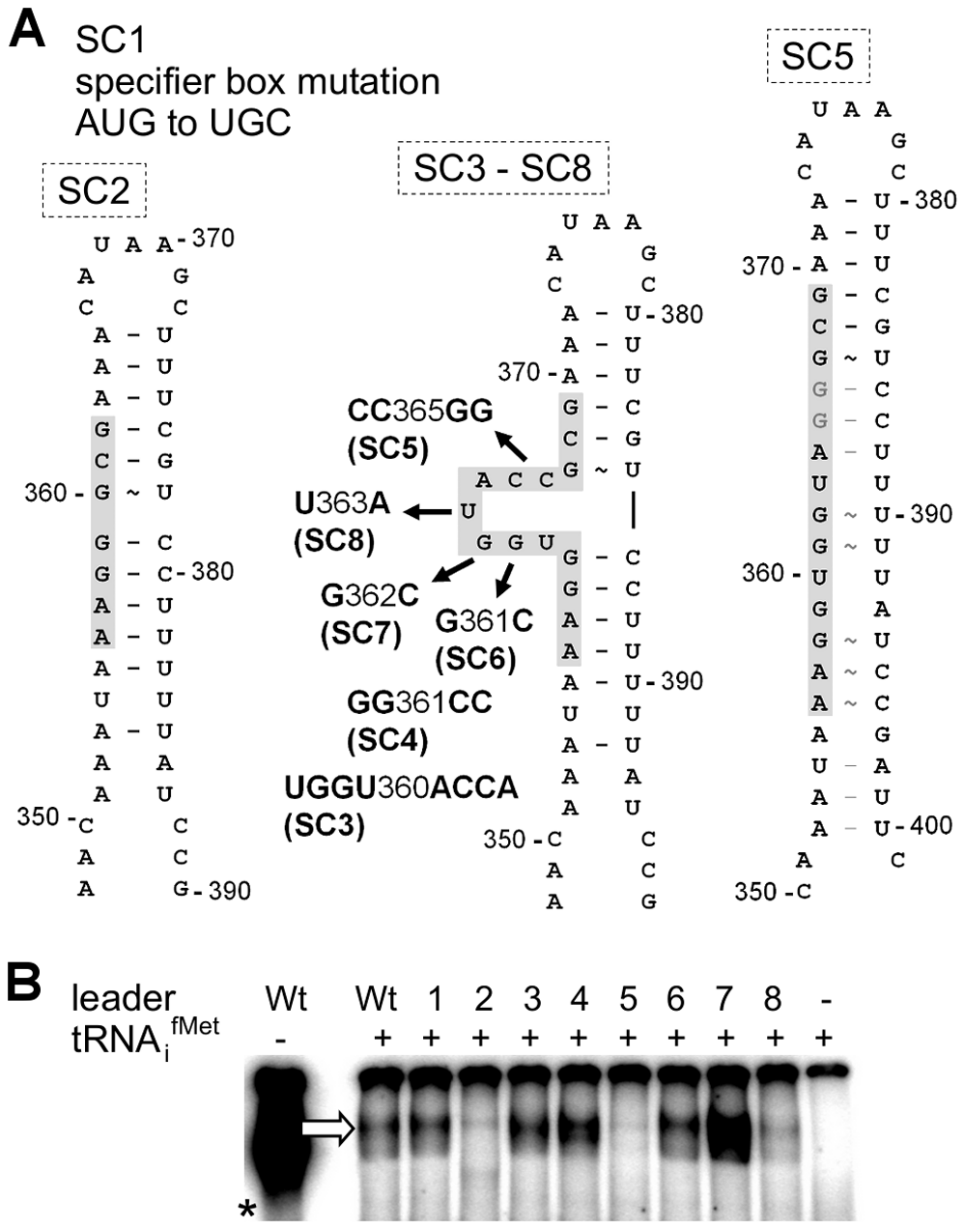
Effect of single nucleotide exchanges in the T-box on tRNA binding. (A) Overview on mutations introduced into the *met* leader RNA. SI Table S2 lists all mutated constructs. The model of the antiterminator structure with the conserved T-box (in grey) demonstrates deletion of the side bulge in SC2, nucleotide substitutions of constructs SC3 to SC8 and the possible loss of the side bulge in SC5 through alternative base pairing. Nucleotide positions refer to the complete length of the *met* leader RNA of *S. aureus* COL. (B) IVT with WT or mutated constructs SC1 to SC8 (lanes 1–8) of the *met* leader RNA template with radiolabeled tRNA_i_^fMet^ present. The arrow indicates the bound tRNA_i_^fMet^. The asterisk marks the control IVT reaction of the WT *met* leader RNA transcribed without tRNA, but in the presence of [α^32^P]-CTP. Results are representative of two independent experiments.

In classical T-box riboswitches, tRNA/leader RNA interaction is mediated by the T-box motif which forms a bulge that facilitates basepairing interactions with the tRNA 3′-CCA and supports antiterminator formation [9] (Figure 2A). We therefore studied whether the predicted T-box motif participates in tRNA_i_^fMet^ binding by generating a series of *met* leader RNAs carrying T-box mutations (SI Table S2, Figure 3A). tRNA_i_^fMet^ binding was clearly diminished in mutants SC2 and SC5, which are both likely to lack the putative T-box bulge for tRNA 3′-CCA interaction (Figure 3A). Also, in SC8, a single U to A exchange at position 363 was sufficient to reduce tRNA_i_^fMet^ binding, whereas other mutations within the putative T-box bulge, *i.e.* SC3, SC4, SC6 and SC7, enhanced tRNA_i_^fMet^ interactions with the *met* leader RNA (Figure 3B). Finally, alteration of a methionine-specific codon AUG to cysteine UGC in the putative specifier box of the *met* leader RNA in SC1 did not affect tRNA_i_^fMet^ binding efficiency (Figure 3B), nor did it confer tRNA^Cys^ binding activity (SI Figure S3) suggesting that other components of this T-box system confer specificity to initiator tRNA_i_^fMet^ binding. Taken together, these data suggest that the *met* leader RNA upstream of the staphylococcal *metICFE-mdh* operon harbors a canonical T-box riboswitch that specifically binds uncharged initiator tRNA_i_^fMet^.

### Methionine starvation induces *met* leader RNA/*metICFE*-*mdh* transcription

**Figure 4:**
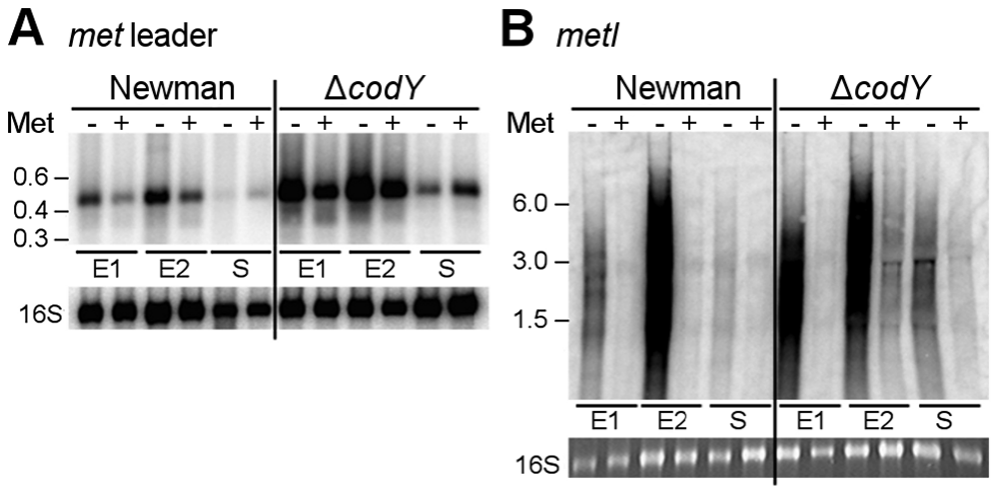
Role of CodY in *met* leader RNA/*metICFE-mdh* transcription control. Northern blot analysis with radiolabeled probes. Total RNA from *S. aureus* strain Newman and its isogenic *codY* mutant was sampled at three different points of growth: early exponential (E1), exponential (E2) and early stationary phase (S). Cultures were grown in CDM without (‘−’) or supplemented with 1 mM L-methionine (‘+’). (A) Hybridization with the *met* leader RNA-specific probe (low range RNA ladder (Fermentas) indicated on the left). The blot was re-hybridized with a 16S rDNA-specific probe, for loading control. (B) Hybridization with *metI*-specific probe (high range RNA ladder (Fermentas) indicated on the left). The 16S rRNA signal on the gel is shown below. Results are representative of two independent experiments.

Through interaction with either uncharged tRNAs (antiterminator formation) or charged tRNAs (terminator formation), T-box riboswitches indirectly sense amino acid levels in the bacterial cell [6], [9]. To determine whether or not *met* leader RNA/*metICFE-mdh* transcription is sensitive to methionine availability, *S. aureus* strain Newman was grown in chemically defined medium (CDM) in the presence or absence of methionine. RNA was isolated in the early exponential (E1), exponential (E2) and early stationary (S) growth phase and analyzed by Northern hybridization using *met* leader RNA- and *metI*-specific DNA probes, respectively (Figure 4A, B). In the presence of methionine, basal *met* leader RNA transcription could be detected. Methionine starvation induced the transcription of this RNA, especially during exponential growth (Figure 4A). In contrast, the *metI* mRNA signal was not detectable in the presence of methionine (Figure 4B). Upon methionine deprivation, however, *metI* mRNA transcription was activated with the strongest expression detected during exponential growth (Figure 4B). Interestingly, *metI*-specific Northern probing did not reveal a distinct fragment representing the full-length *metICFE-mdh* mRNA (Figure 4B). Instead, an RNA smear was detected in repeated experiments. As the RNA quality and integrity had been confirmed prior to the experiments, the Northern blot data suggest rapid degradation of the methionine starvation-induced *metICFE-mdh* transcript.

### CodY controls *met* leader RNA transcription in response to methionine supply

CodY is a global transcription repressor that controls the expression of a variety of genes in *S. aureus*, many of which being involved in amino acid biosynthesis and transport [10], [11]. Detection of a consensus sequence for CodY binding upstream of the *met* leader RNA and the recent identification of the methionine biosynthesis genes as direct CodY targets in *S. aureus* [10] prompted us to study the role of this factor for *met* leader/*metICFE-mdh* transcription in more detail. A *S. aureus codY* deletion mutant was grown in CDM with and without methionine and analyzed for *met* leader and *metICFE-mdh* transcription by Northern blot hybridization. In the absence of methionine, a stronger *met* leader RNA signal was detected in both the wildtype and the *codY* mutant (Figure 4A). However, compared to the wildtype, the *codY* mutant showed a generally enhanced *met* leader transcription, suggesting that *met* leader RNA expression was de-repressed in the absence of CodY irrespective of whether methionine was present or not (Figure 4A). In contrast, downstream *metICFE-mdh* operon expression remained sensitive to varying concentrations of methionine and was only activated both in the wildtype and the *codY* mutant in the absence of methionine (Figure 4B).

### Methionine depletion is signaled to CodY via the stringent response

**Figure 5:**
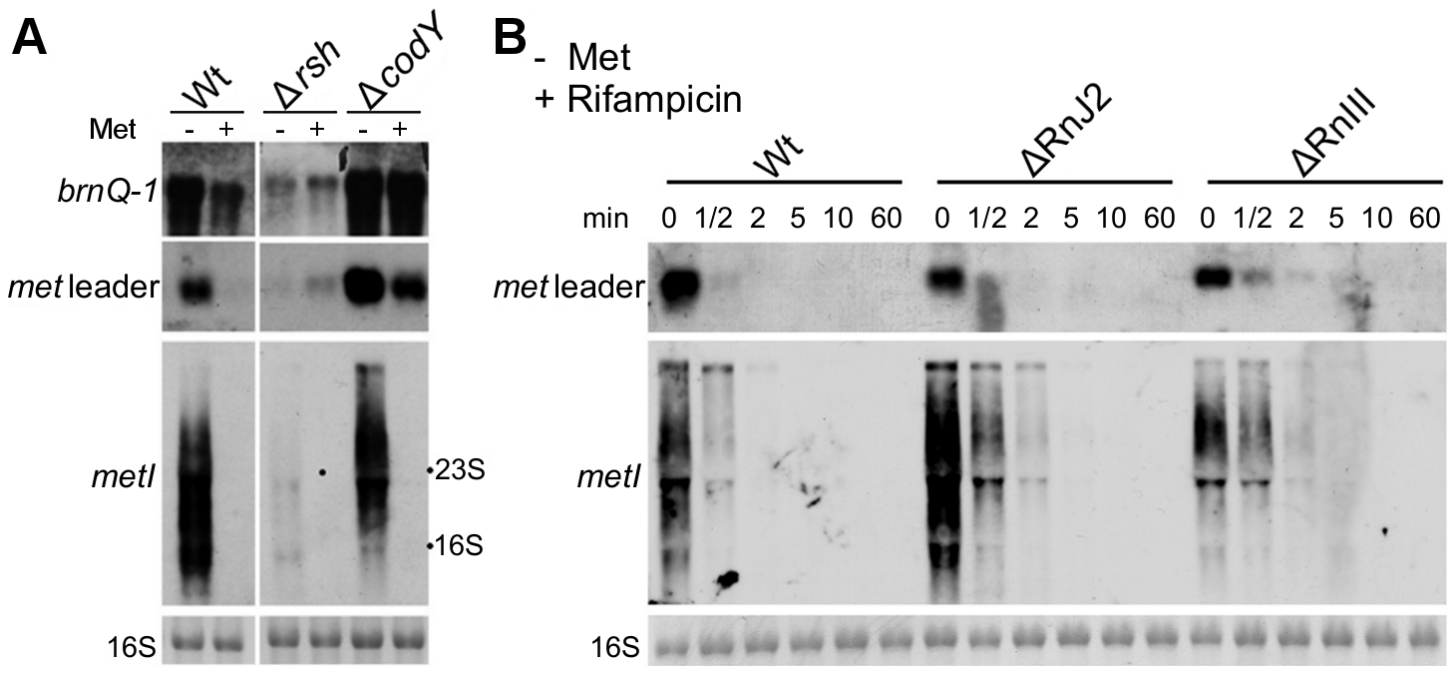
Stringent response relay and involvement of RNases. (A) Northern blot analysis of total RNA from *S. aureus* strain Newman (‘Wt’) and different isogenic mutants (‘Δ’) sampled at exponential growth phase. The cultures were grown in CDM without (‘−’) or supplemented with 1 mM L-methionine (‘+’). DIG-labeled DNA probes for *brnQ-1* (branched-chain amino acid transport), the *met* leader RNA or *metI* were used to hybridize RNA from WT, the *rsh* (ppGpp synthetase) and a *codY* mutant. The data are representative of two independent experiments. (B) RNA stability assay. Northern blot analyses of *met* leader RNA (upper panel) and *metICFE-mdh* mRNA (lower panel) upon rifampicin exposure of *S. aureus* strain Newman (‘Wt’) and isogenic RNase J2 and RNase III deletion mutants (‘ΔRnJ2’, ‘ΔRnIII’), respectively. Bacteria were grown in CDM without methionine and total RNA was prepared from cells before (0 min) and after the addition of 500 µg ml^−1^ rifampicin at the time points indicated in the figure. Blots were hybridized with DIG-labeled *met* leader- and *metI*-specific DNA probes, respectively. Bottom panels in (A) and (B) show the 16S rRNA as loading controls in the corresponding agarose gels.

In many bacteria, nutrient limitation triggers the so-called stringent response to appropriately adjust gene expression patterns. Stringent response is characterized by the rapid synthesis of the alarmone (p)ppGpp involving bifunctional RelA/SpoT synthetases/hydrolases (RSHs) and affecting/modulating many cellular functions. Recently, a link between CodY and the stringent response of *S. aureus* has been demonstrated [12]. The CodY repressor function depends on its two effector molecules GTP and branched-chain amino acids (BCAA, valine, leucine, isoleucine) which enhance synergistically the affinity of CodY for its DNA targets [13], [14]. RSH-mediated (p)ppGpp synthesis lowers the GTP levels in the cell and eventually facilitates release of CodY from its DNA targets [12]. In a next set of experiments, we sought to identify possible regulatory links between methionine deficiency, stringent response and CodY. For this purpose, *met* leader RNA/*metICFE-mdh* transcription upon methionine depletion was studied in a *rsh* mutant carrying a deletion of the (p)ppGpp synthetase domain in strain *S. aureus* Newman [12]. As a marker for stringent response-controlled genes, *brnQ-1*, which encodes a CodY-repressed BCAA permease, was included in the analysis. In the *S. aureus* wildtype, methionine depletion led to *brnQ-1* induction along with *met* leader RNA and *metICFE-mdh* expression (Figure 5A). In contrast, in the *rsh* mutant, induction of both *brnQ-1* and *met* leader/*metICFE-mdh* transcription were significantly reduced upon methionine starvation in comparison to the wildtype, suggesting that RSH-mediated (p)ppGpp synthesis may be required for efficient activation of the system. Deletion of *codY* resulted in a generally higher basal transcription of both *brnQ-1* and *met* leader RNA in the presence of methionine, whereas *metICFE-mdh* transcription remained tightly controlled and switched off under these conditions (Figure 5A). In the *codY* mutant, *brnQ*-1 expression was de-repressed and not further inducible by methionine deprivation, suggesting that CodY is responsible for the methionine-dependent *brnQ-1* induction observed in the wildtype (Figure 5A).

### RNase J2 and RNase III participate in *met* leader RNA and *metICFE*-*mdh* mRNA decay

The experiments described in Figure 4 suggest that *metICFE-mdh* mRNA is subject to rapid degradation. To investigate the possible involvement of specific RNases in this process, the stability of *met* leader RNA and *metICFE-mdh* was analyzed in *S. aureus* mutants that were deficient in RNase J2 and RNase III activity, respectively. For this purpose, *de novo* RNA synthesis was interrupted by the addition of the RNA polymerase inhibitor rifampicin to the cultures. Total RNA was isolated at different time points and subjected to Northern blot analysis (Figure 5B). Comparison of the wildtype and the RNase-deficient mutants revealed that the *metICFE-mdh* transcript was more stable in the RNase J2 mutant, suggesting that RNase J2 may be involved in *metICFE-mdh* degradation. In contrast, no significant effect on *met* leader RNA stability was detectable in the RNase J2 mutant (Figure 5B, upper panel). The RNase III mutant exhibited a slightly enhanced stability both of the *met* leader and *metICFE-mdh* RNA indicating a possible function of RNase III in *met* leader RNA and *metICFE-mdh* decay (Figure 5B).

## Discussion

### The staphylococcal *met* leader RNA interacts with initiator formylmethionyl-tRNA

In this study, we show that methionine biosynthesis control in *S. aureus* involves a T-box riboswitch. While the conservation of this T-box in staphylococci was predicted previously using bioinformatic tools [5], [6], [8], we now provide, for the first time, direct experimental proof for a specific interaction of the predicted T-box with initiator tRNA_i_^fMet^. While methionyl-tRNA-specific T-box riboswitches (met-T-box) are rare among *Bacillales* they are more common in *Lactobacillales*. In both orders they are associated with methionine metabolism or transport (Table 1). Methionyl-tRNAs (tRNA^Met^) are encoded by four distinct gene loci in the genomes of *S. aureus* and *S. epidermidis*. Two of them are identical and represent the initiator tRNA_i_^fMet^, while the other two tRNA^Met^ loci differ in their nucleotide sequence from each other and from the tRNA_i_^fMet^. Surprisingly, we found a clear preference for interaction of the *met* leader RNA T-box with the initiator tRNA_i_^fMet^ (Figure 2B). In prokaryotes, the first N-terminal methionine of newly synthesized proteins is N-formylated and, hence, N-formylmethionine (fMet) is indispensable for protein translation initiation and bacterial growth. fMet is carried to the ribosomal translation initiation complex by tRNA_i_^fMet^ which differs structurally from the elongator tRNA^Met^ used for the incorporation of methionine residues into the growing polypeptide chain. Although all tRNA^Met^ are charged with methionine by (the same) methionyl-tRNA synthetase, it is only tRNA_i_^fMet^ that is specifically recognized by the methionyl-tRNA-formyltransferase which then mediates N-formylation of methionine to produce fMet. At present, it is not clear how the observed specificity of the *met* leader RNA T-box for tRNA_i_^fMet^ is accomplished. An involvement of the putative specifier box in the 5′-region of the *met* leader RNA seems unlikely because all methionyl-tRNAs use the same anticodon, suggesting that other regions of the *met* leader RNA interact with structures that are unique to the initiator tRNA_i_^fMet^. In line with this hypothesis, our data showed that tRNA_i_^fMet^ binding by the *met* leader RNA was not affected when the specifier box in the *met* leader was substituted with a cysteine-specific codon (Figure 3B). Also, these nucleotide replacements were insufficient to confer tRNA^Cys^ binding activity (SI Figure S3). Our observation that the T-box riboswitch, shown in this study to be a key regulator of methionine biosynthesis in *S. aureus*, preferentially binds tRNA_i_^fMet^ points to an elegant mechanism by which protein translation initiation efficiency could both be sensed and, if necessary, adjusted by modulating fMet supply. It will be interesting to investigate if this tRNA_i_^fMet^ preference also applies to other met-T-box riboswitches that control the expression of genes not directly involved in methionine biosynthesis. Also, potential metabolic implications of the use of T-box-controlled fMet supply in staphylococci versus S-box-controlled methionine biosynthesis in other bacteria remain to be studied.

**Table 1:** Methionine metabolism and biosynthesis control among *Bacillales* and *Lactobacillales*.

	Methionine salvage	Polyamine synthesis	SAM recycling	Methionine biosynthesis control by	Number of predicted methionine-specific T-box riboswitches
***Bacillales***					
*Bacillus subtilis*	**+**	**+**	**+**	CodY- S-box	0
*Bacillus cereus*	**+**	**+**	**+**	S-box	1 (Methionyl-tRNA synthetase *metS*)
*Listeria monocytogenes*	**−**	**(+)**	**+**	S-box	0
*Oceanobacillus iheyensis*	**−**	**+**	**+**	CodY-S-box	0
*Staphylococcus aureus*	**−**	**−**	**+**	CodY- T-box	1 (*metICFE*-*mdh*)
***Lactobacillales***					
*Lactobacillus plantarum*	**−**	**−**	**+**	T-box	7 (methionine synthesis & transport)
*Lactobacillus delbrueckii*	**−**	**−**	**+**	T-box	3 (methionine synthesis & transport, oligopeptide transport)
*Lactobacillus casei*	**−**	**−**	**+**	T-box	4 (methionine synthesis & transport)
*Leuconostoc mesenteroides*	**−**	**−**	**+**	T-box	6 (methionine synthesis & transport)
*Enterococcous faecalis*	**−**	**−**	**+**	no methionine biosynthesis	4 (methionine transport)

Presence of genes of the methionine salvage pathway, polyamine synthesis, SAM recycling and control mechanisms of methionine biosynthesis in genomes of bacteria of the *Bacillales* and *Lactobacillales* orders as well as number of methionine-specific T-box riboswitches in these genomes. Data were obtained by querying the KEGG http://www.genome.jp/kegg/ and RegPrecise http://regprecise.lbl.gov/RegPrecise/index.jsp databases [15] and from references [6], [20].

### T-box-controlled methionine biosynthesis is linked to stringent response and CodY regulation

**Figure 6:**
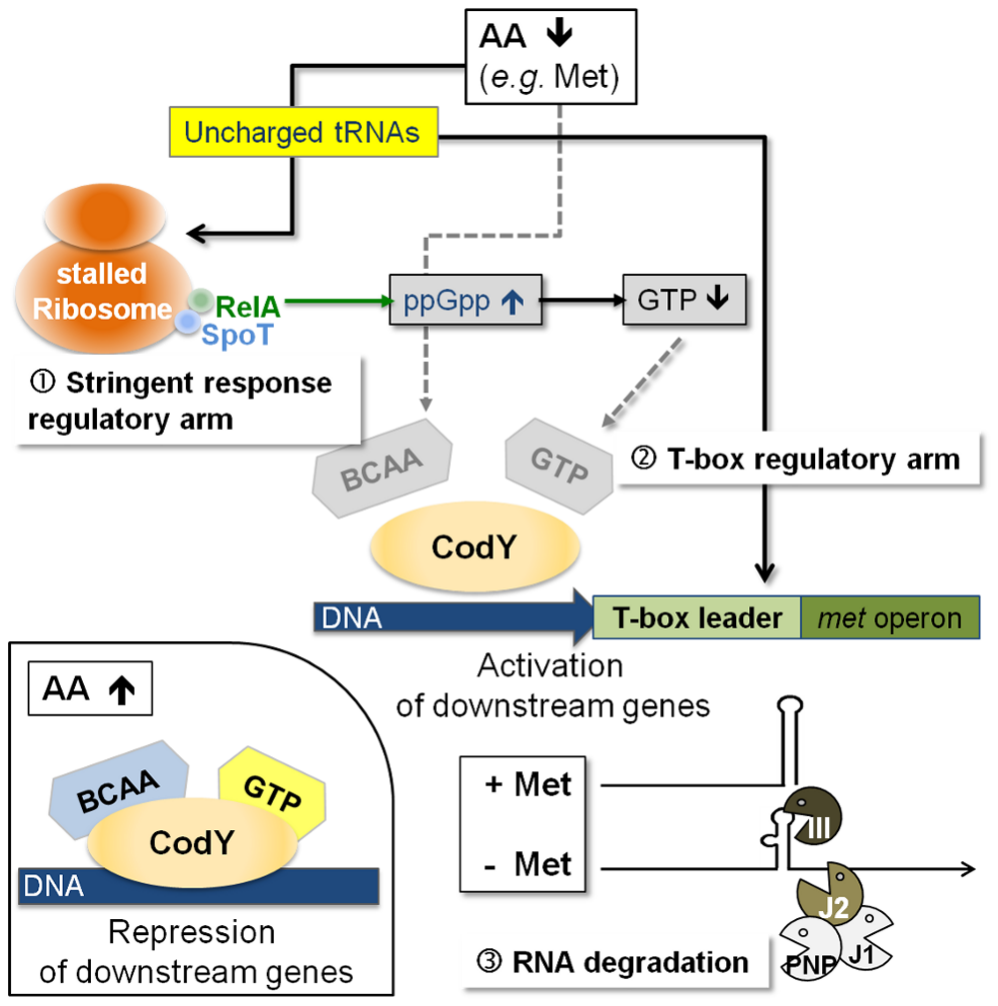
Model of a regulatory cascade for methionine biosynthesis operon control. (i) With high amino acid levels, branched-chain amino acids (BCAA) and GTP are bound to the CodY repressor, increasing its affinity for target DNA binding; downstream genes are repressed (small picture, bottom left). (ii) Low amino acid levels will trigger 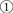 the stringent response due to stalled ribosomes, which leads to an increase in RelA-mediated ppGpp alarmone synthesis resulting in less GTP. Subsequently, CodY dissociates from the DNA activating downstream transcription of the T-box leader RNA. 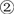 The T-box acts as the crucial check-point sensing uncharged tRNA_i_^fMet^ levels and determines transcription of the *met* biosynthesis genes in a highly methionine-dependent manner. 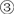 Rapid degradation of the *met* mRNA by the RNA degradosome is an additional mechanism to limit unnecessary translation of methionine biosynthesis mRNA.

The data obtained in this study lead us to propose that a hierarchical regulatory network controls methionine biosynthesis in *S. aureus*, most likely, to minimize unnecessary *de novo* methionine biosynthesis. Centerpiece of this regulation turns out to be the tRNA_i_^fMet^-specific T-box riboswitch located in the 5′-*met* leader that precedes the coding regions of the *metICFE-mdh* mRNA. Another important player is the global repressor CodY which drives *met* leader RNA transcription and links the system to the metabolic emergency circuit of the bacterial stringent response. Finally, staphylococcal RNases were implicated in this network by degrading both *metICFE-mdh* mRNA and *met* leader RNA, which may be considered a form of posttranscriptional control of *metICFE-mdh* gene expression. Figure 6 summarizes our major findings and suggests a model for the control of methionine biosynthesis in staphylococci. While stringent response-mediated CodY release and subsequent *met* leader RNA transcription are sensitive to general amino acid availability and the energy status of the cell, the T-box riboswitch is highly selective and ensures that downstream *metICFE-mdh* mRNA transcription only occurs if methionine concentration is low. The experiments also indicate that lack of methionine alone is sufficient to trigger the stringent response and, as a consequence, the release of CodY, thus securing efficient *met* leader RNA/*metICFE-mdh* transcription when needed (Figure 6). Interestingly, the regulatory cascade identified in this study seems to represent an exception rather than common rule. Thus, database searches of *B. subtilis* and *S. aureus* genomes revealed that combinations of CodY with T-box riboswitches are restricted to methionine and tryptophan biosynthesis in *S. aureus* and branched-chain amino acid (BCAA) biosynthesis in *B. subtilis*, respectively [15], [16] (Table 1).

### RNA decay is another checkpoint in methionine biosynthesis control

Methionine biosynthesis is a costly process consuming ATP and other resources. Riboswitches are generally regarded as fast and tight regulatory systems that do not depend on protein factors which, in many cases, react more slowly and/or are subject to complex regulation of protein expression, activation and degradation [5], [17]. Our study suggests that, in addition to regulation at the transcriptional level, *S. aureus* employs RNA decay mechanisms to quickly remove newly transcribed *metICFE-mdh* mRNA from the system, thus further limiting the risk of sustained (over)expression of genes involved in methionine synthesis. Although the complete mechanism and enzymes involved in the process still need to be established, the data give a first hint that RNase J2 participates in *metICFE-mdh* degradation (Figure 5B). This is in contrast to the situation in *B. subtilis* where mRNAs of the methionine biosynthesis genes, polyamine synthesis as well as the methionine salvage pathway (see below) were found to be not degraded by RNase J1/J2 [18]. The data further suggest that RNase III might be involved in *met* leader RNA degradation (Figure 5B). This observation is consistent with previously published data showing that RNase III co-immunoprecipitates with *met* leader RNA and targets also other *S. aureus* riboswitches for degradation [19]. Taken together, the data lead us to suggest that RNA decay is another mechanism involved in the control of methionine synthesis in staphylococci that merits further future investigation.

### Why do staphylococci use a T-box riboswitch for methionine biosynthesis control?

T-box control of methionine biosynthesis genes in staphylococci is an exception among *Bacillales* which usually regulate this pathway by S-adenosylmethionine (SAM)-binding S-box riboswitches [5]. Apart from protein synthesis, most microorganisms use methionine to produce SAM which plays a central role in many cellular functions [4]. First, SAM serves as a methyl group donor for nucleic acid and protein methylation. Products of the methylation reaction are detoxified and recycled to homocysteine which is then reused for methionine/SAM synthesis. Second, SAM is used, following decarboxylation, to form polyamines. The remaining 5′-methylthioadenosine moiety is again metabolized to methionine by enzymes of the methionine salvage pathway. Comparative genomics using the KEGG database (http://www.genome.jp/kegg/) and experimental research [20], [21], [22] suggest that, unlike *Bacillus*, staphylococci may have only limited capacity to reuse or redirect methionine to other pathways because they lack both the methionine salvage and polyamine synthesis pathways (Table 1). Therefore, synthesis and recycling of SAM may be the only possibility to make use of excess methionine, implying that a more stringent control of *de novo* biosynthesis may be required in staphylococci. Interestingly, the *Lactobacillales*, which preferentially control their methionine biosynthesis genes by T-box riboswitches [5], also appear to lack polyamine synthesis and methionine salvage genes (Table 1). Based on these observations, we hypothesize that the lack of both polyamine synthesis and methionine salvage might favor control by a T-box rather than a S-box riboswitch, the major advantage being that the T-box riboswitch is able to sense methionine supply directly and then react immediately by switching off transcription of methionine biosynthesis genes, whereas S-box regulated systems would require an additional step (*i.e.* SAM synthesis) to produce the effector molecule required to stop methionine production. Alternatively, microorganisms that produce polyamines may need a larger SAM pool as precursor for the synthesis of these important compounds. Therefore, S-box control of methionine biosynthesis might be more effective in these organisms to ensure a constant SAM supply. More experimental work is needed to further substantiate these hypotheses.

### The methionine pathway - a suitable anti-staphylococcal drug target?

The general life style of staphylococci provides easy access to methionine sources from the respective host they colonize or infect and, therefore, suggests that methionine supply may not be a limiting factor under normal conditions. The data presented in this paper provide first insight into the regulation of methionine synthesis gene expression in staphylococci and, more importantly, show that staphylococci have evolved special mechanisms to tightly restrict *de novo* methionine biosynthesis. It is tempting to speculate that overproduction (rather than lack) of methionine may be critical to staphylococci and, thus, this strict control of methionine *de novo* synthesis would not only save resources and energy but also meet the requirement to prevent methionine accumulation. While excess methionine biosynthesis might be critical for staphylococci under most conditions, *de novo* methionine biosynthesis becomes crucial for growth and survival when methionine supply is limited, for example, when entering the host during infection [23] or under specific external stress conditions like antibiotic and antimicrobial peptide exposure [24], [25]. It is therefore conceivable that both the efficient activation and shut-off of methionine biosynthesis might represent a metabolic “Achilles' heel” for staphylococci. Interestingly, successful inhibition of the *S. aureus* methionyl-tRNA synthetase by an experimental compound has already provided evidence that methionyl-tRNA metabolism is a suitable anti-staphylococcal target [26]. Also, structure-based drug design recently resulted in the identification of lead compounds that specifically interact with T-box structures *in vitro*, indicating that RNA-based drug targeting is a promising new avenue in medicinal chemistry [27], [28], [29]. The data presented in this paper may open perspectives for specifically targeting methionine metabolism and protein translation initiation in future efforts to develop novel *Staphylococcus*-specific antibiotics.

## Materials and Methods

Bacterial strains, construction of mutants, growth conditions as well as RNA isolation and Northern blot procedures can be found in the SI Materials and Methods (Text S1, Table S1–S5).

### 3′ and 5′ RACE experiments

Total RNA was treated with recombinant DNase I (Ambion) and RNA quality was checked with the Agilent 2100 Bioanalyzer (Agilent Technologies). The 5′/3′ RACE Kit, 2^nd^ Generation (Roche) was used for determination of transcript ends by synthesis of first-strand cDNA with reverse transcriptase (Fermentas) and the oligonucleotides listed in Table S4 (Text S1).

### Site directed mutagenesis of the *met* leader RNA template

The *met* leader RNA sequence of *S. aureus* COL was amplified by PCR with oligonucleotides T7-F_met-sRNA and R_met-sRNA (Tables S5, Text S1). The product was inserted into the pGEM-T Easy vector (Promega) yielding plasmid pGEM*met*COL. For site directed mutagenesis, the oligonucleotides listed in Table S5 were used in PCR reactions with pGEM*met*COL as a template followed by *Dpn*I treatment prior to transformation of the PCR products into *Escherichia coli* DH5α cells. The rescued plasmids were sequenced and constructs SC1 to SC8 (Table S2) with the appropriate mutations were used as *met* leader RNA templates in tRNA binding assays.

### *In vitro* transcription (IVT) and tRNA binding assay

tRNA templates were generated by PCR from genomic DNA of *S. aureus* COL and oligonucleotides listed in Table S4 (Text S1). Four picomol DNA template were subsequently used for IVT in a 20 µl reaction volume consisting of T7 transcription buffer, 20 U RNase inhibitor, 20 U T7 RNA polymerase (Fermentas), 0.5 mM ATP, UTP, GTP, 12 µM CTP and 9 mM GMP as described [30]. [α^32^P]-CTP was added and the reaction was incubated at 37°C for six hours. For *met* leader RNA *in vitro* transcription, PCR templates were either generated from plasmid pGEM*met*COL or constructs SC1 to SC8 (Table S2) with oligonucleotides T7-F_met-sRNA and R_met-sRNA (Table S5). The products were used in IVT/tRNA binding assays in the presence of pre-formed tRNAs: 10 µl reaction volume consisted of T7 transcription buffer, 10 U RNase inhibitor, 6 U T7 RNA polymerase (Fermentas) and 0.5 mM NTPs. DNA templates of the *met* leader RNA and of pre-formed tRNA were adjusted to end concentrations of 8 nM and 50 nM, respectively, and the mix was incubated at 37°C for two hours. Samples were immediately separated on a non-denaturing 6% (w/v) polyacrylamide gel by electrophoresis at 4°C. Visualization was attained with the PhosphoImager (Fujifilm FLA-7000) and quantification of bands was achieved with the software Multi Gauge V2.2.

### Rifampicin assay for determination of transcript stability

Bacteria from overnight cultures were diluted in 100-ml flasks in 40 ml CDM medium with methionine to an initial optical density at 600 nm (OD_600_) of 0.05 and grown with shaking at 220 rpm at 37°C to an OD of 0.5. The cultures were filtered over a 0.22 mm filter applying vacuum, washed twice with sterile phosphate buffered saline (PBS) and bacteria were resuspended in 15 ml CDM medium without methionine and grown for another 60 minutes in a 30-ml tube. Then rifampicin (500 µg ml^−1^) was added to the cultures. Before (0) and after 0.5, 2, 5, 10 and 60 minutes of rifampicin exposure, RNA was isolated and Northern blot analyses were performed as described in the Supporting Information (Text S1).

## Supporting Information

Figure S1 **Complete sequence alignment of the intergenic region of the** ***met*** **leader RNA.** The two *Staphylococcus epidermidis* genomes as well as seven *S. aureus* genomes were aligned with the genomes of *S. haemolyticus* and *S. saprophyticus* for the intergenic region between SACOL0431 and SACOL0432. The multiple sequence alignment was done with ClustalW2 [3] on the server of the European Bioinformatics Institute (http://www.ebi.ac.uk/Tools/msa/clustalw2/). Known motifs are highlighted. Purple boxes contain conserved motifs identified in *B. subtilis* T-box systems [4].
(TIFF)

Figure S2 **Structural model of the full-length** ***S. epidermidis*** **RP62A** ***met*** **leader RNA.** The secondary structure model of the *met* leader RNA was based on the structural determination of the *B. subtilis tyrS* T-box leader RNA [4], [5]. Nucleotides conserved among staphylococcal genomes (see alignment Figure S1) are highlighted in blue, those conserved in *B. subtilis* T-box systems are indicated by asterisk. The first hairpin structure from nucleotides 20 to 125 includes several conserved structural motifs described for the *B. subtilis tyrS* T-box leader RNA, such as the GA-motif at the base, the AG-bulge and the S-turn motif opposite the potential specifier loop as well as the conserved sequence in the apical loop of stem I [4], [5]. Among staphylococcal genomes, the region from nucleotides 200 to 350 contains regions of variable length and structure (green boxes). The mutually exclusive structure of terminator and antiterminator hairpin from nucleotide 375 till the 3′ end harbors highly conserved sequences (see Figure S1) with only compensatory mutations in the stem structure or in the apical loops. The T-box motif itself (position 378–391) is 100% conserved among the staphylococcal genomes and forms the T-box side bulge within the antiterminator structure.
(TIFF)

Figure S3 ***In vitro*** **binding assay of tRNA^Cys^ with SC1.** *In vitro* transcription of the *met* leader RNA template was done in the presence of either tRNA_i_^fMet^ or tRNA^Cys^. On the left the wildtype sequence of the *met* leader RNA was used, on the right the construct SC1 with mutation of the specifier box from methionine (AUG) to cysteine (UGC) codon. The asterisk indicates the control reaction without tRNA present but [α^32^P]-CTP. The arrow indicates the binding interaction of tRNA_i_^fMet^ with the *met* leader RNA.
(TIFF)

Text S1 **Additional material and methods information as well as Tables S1 to S5.** Descriptions are given of the experimental setup concerning cell growth and viability in chemically defined medium, the construction of conditional RNase J2 (*rnjB*) and RNase III (*rnc*) mutants and details of the RNA isolation and Northern blot analysis protocols used here. Tables S1 to S5 list the bacterial strains and plasmids as well as all oligonucleotides used in this study.
(PDF)
