# Supplementary figures and images for: Methionine Biosynthesis in Staphylococcus aureus Is Tightly Controlled by a Hierarchical Network Involving an Initiator tRNA-Specific T-box Riboswitch
Source: PLoS Pathog. 2013 Sep 12;9(9):e1003606. doi: 10.1371/journal.ppat.1003606 (PMC3771891; doi:10.1371/journal.ppat.1003606)

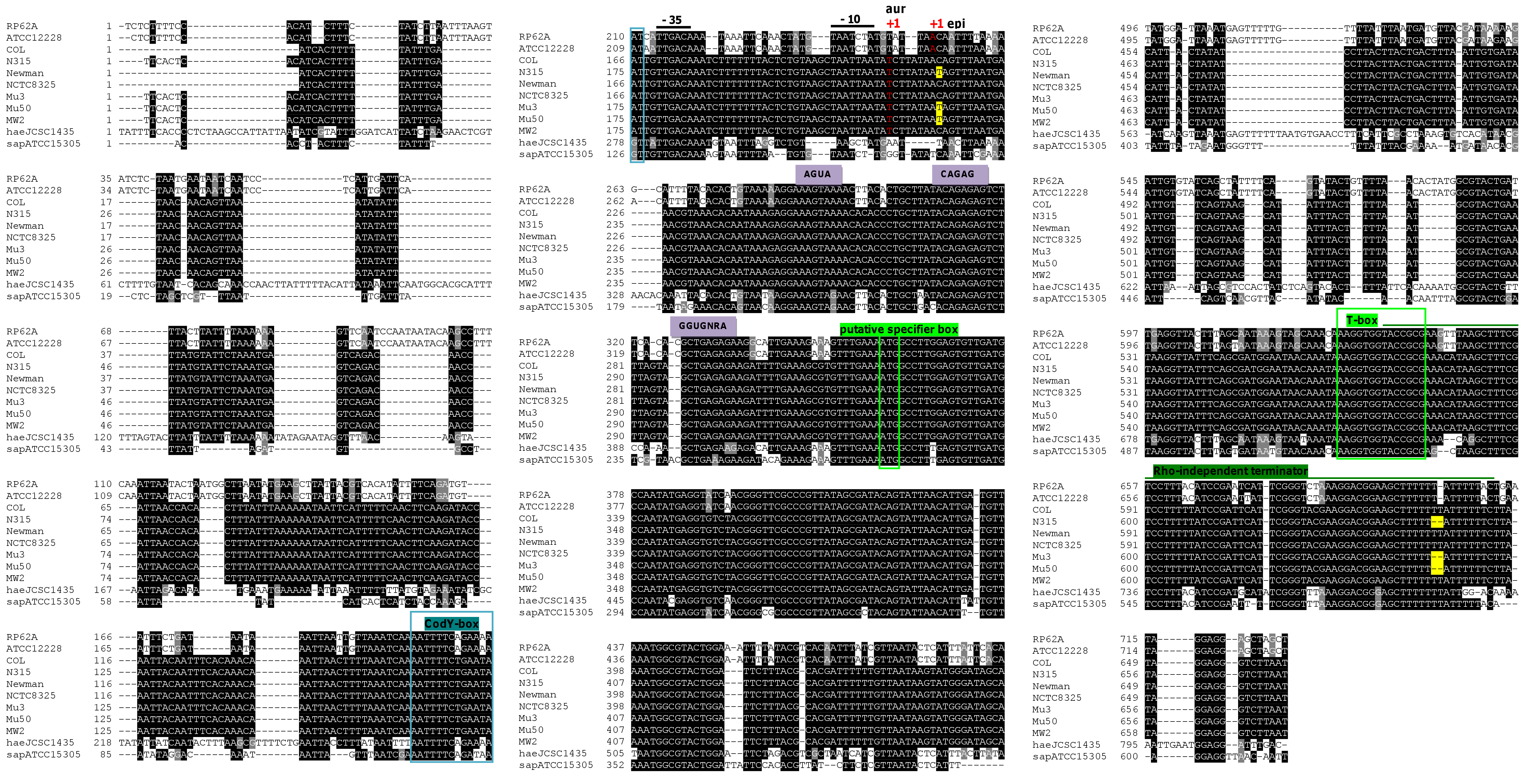

Supplement: Figure S1 — Complete sequence alignment of the intergenic region of the met leader RNA. The two Staphylococcus epidermidis genomes as well as seven S. aureus genomes were aligned with the genomes of S. haemolyticus and S. saprophyticus for the intergenic region between SACOL0431 and SACOL0432. The multiple sequence alignment was done with ClustalW2 [3] on the server of the European Bioinformatics Institute (http://www.ebi.ac.uk/Tools/msa/clustalw2/). Known motifs are highlighted. Purple boxes contain conserved motifs identified in B. subtilis T-box systems [4]. (TIFF) [file ppat.1003606.s001.tiff]

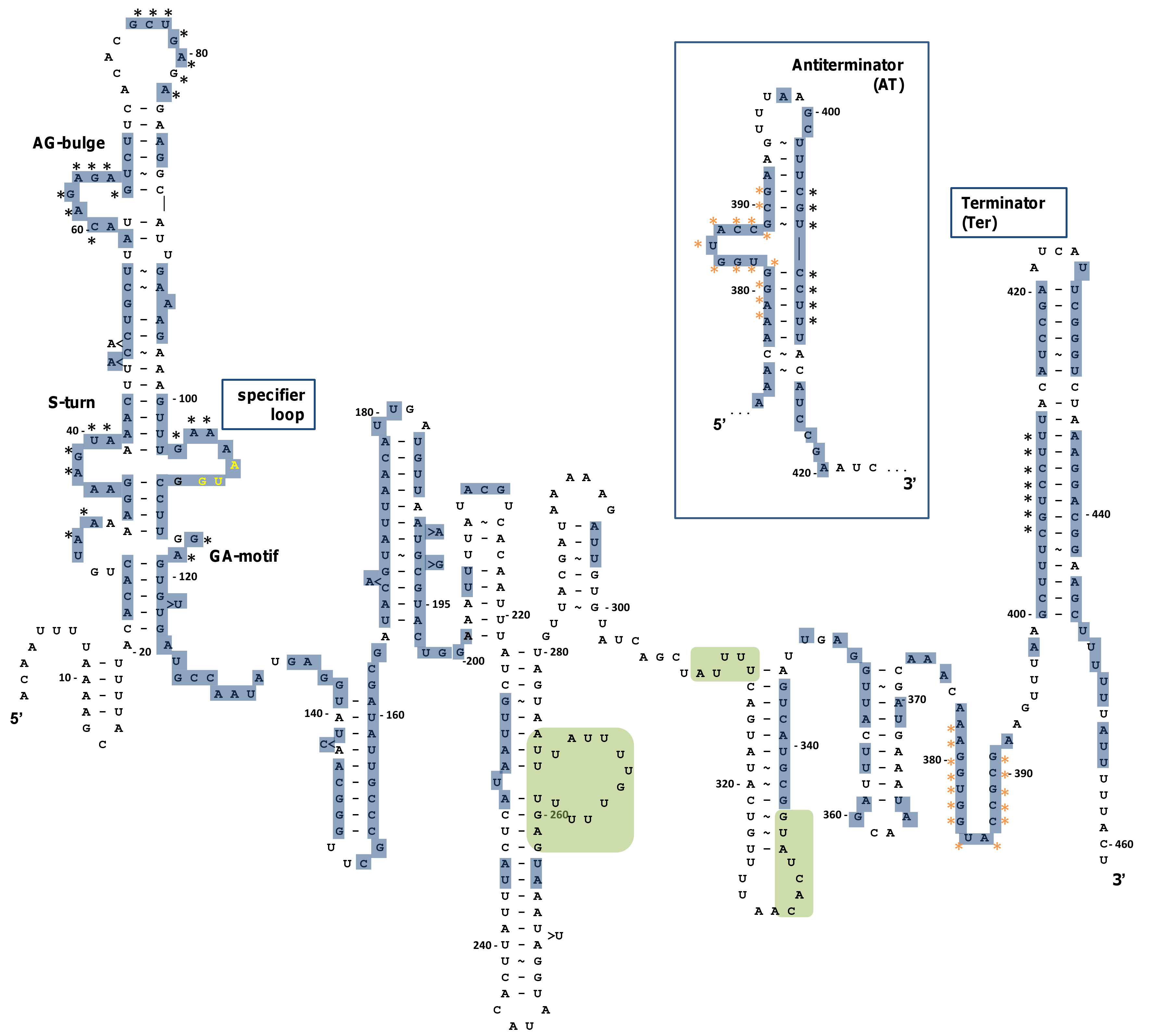

Supplement: Figure S2 — Structural model of the full-length S. epidermidis RP62A met leader RNA. The secondary structure model of the met leader RNA was based on the structural determination of the B. subtilis tyrS T-box leader RNA [4], [5]. Nucleotides conserved among staphylococcal genomes (see alignment Figure S1) are highlighted in blue, those conserved in B. subtilis T-box systems are indicated by asterisk. The first hairpin structure from nucleotides 20 to 125 includes several conserved structural motifs described for the B. subtilis tyrS T-box leader RNA, such as the GA-motif at the base, the AG-bulge and the S-turn motif opposite the potential specifier loop as well as the conserved sequence in the apical loop of stem I [4], [5]. Among staphylococcal genomes, the region from nucleotides 200 to 350 contains regions of variable length and structure (green boxes). The mutually exclusive structure of terminator and antiterminator hairpin from nucleotide 375 till the 3′ end harbors highly conserved sequences (see Figure S1) with only compensatory mutations in the stem structure or in the apical loops. The T-box motif itself (position 378–391) is 100% conserved among the staphylococcal genomes and forms the T-box side bulge within the antiterminator structure. (TIFF) [file ppat.1003606.s002.tiff]

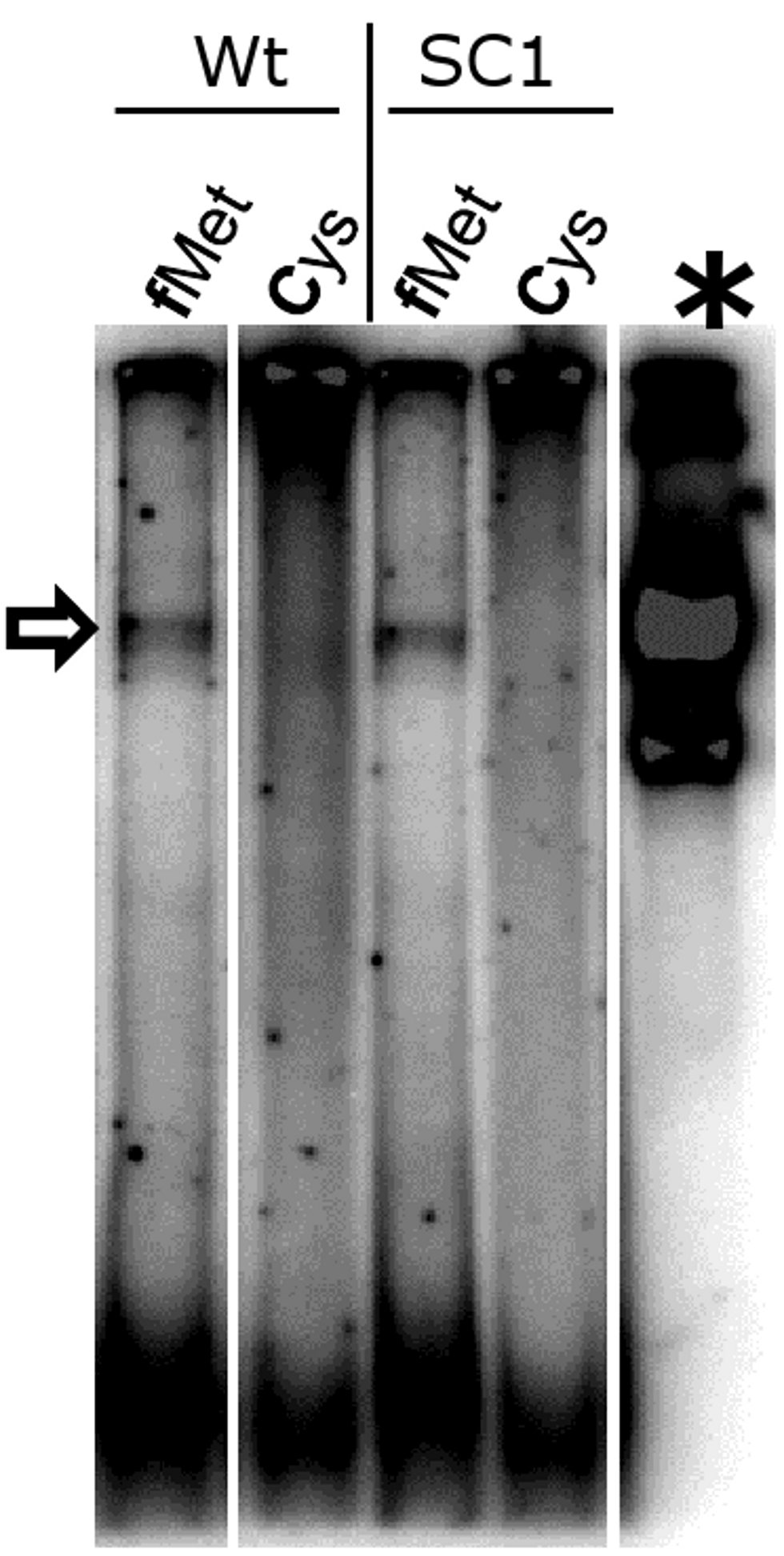

Supplement: Figure S3 — In vitro binding assay of tRNACys with SC1. In vitro transcription of the met leader RNA template was done in the presence of either tRNAi fMet or tRNACys. On the left the wildtype sequence of the met leader RNA was used, on the right the construct SC1 with mutation of the specifier box from methionine (AUG) to cysteine (UGC) codon. The asterisk indicates the control reaction without tRNA present but [α32P]-CTP. The arrow indicates the binding interaction of tRNAi fMet with the met leader RNA. (TIFF) [file ppat.1003606.s003.tiff]
