# Supplementary material for: Methionine Biosynthesis in Staphylococcus aureus Is Tightly Controlled by a Hierarchical Network Involving an Initiator tRNA-Specific T-box Riboswitch
Source: PLoS Pathog. 2013 Sep 12;9(9):e1003606. doi: 10.1371/journal.ppat.1003606 (PMC3771891; doi:10.1371/journal.ppat.1003606)
Supplement: Text S1 — Additional material and methods information as well as Tables S1 to S5. Descriptions are given of the experimental setup concerning cell growth and viability in chemically defined medium, the construction of conditional RNase J2 (rnjB) and RNase III (rnc) mutants and details of the RNA isolation and Northern blot analysis protocols used here. Tables S1 to S5 list the bacterial strains and plasmids as well as all oligonucleotides used in this study. (PDF) [file ppat.1003606.s004.pdf]

## SI Material and Methods

### Monitoring of cell growth and viability

Bacterial strains used are listed in Table S1. *Staphylococcus* strains were grown in either complex medium TSB (tryptone soya broth, Oxoid) or in a chemically defined medium (CDM). The latter consisted of a basic salt medium (12.5 mM $Na_2HPO_4$, 10 mM $KH_2PO_4$, 1.65 mM $MgSO_4$, 9.25 mM $NH_4Cl$, 8.5 mM NaCl), 0.142 mM sodium citrate tribasic dihydrate, 75 mM α-D(+) glucose and 1 mM of all 19 L-amino acids. L-methionine (Met) was only added when indicated. Vitamins and trace elements were supplemented as follows (with final molarity in nM in brackets): cyanocobalamin (108), 4-aminobenzoic acid (870), D-biotin (120), nicotinamide (2430), Ca-D-panthothenic acid (630), pyridoxine hydrochloride (1860), thiamine hydrochloride (870), riboflavin (780), $ZnCl_2$ (510), $MnCl_2$ (500), $H_3BO_3$ (97), $CoCl_2$ (1460), $CuCl_2$ (15), $NiCl_2$ (100), $Na_2MoO_4$ (148) and $FeCl_3$ (750).
For strains carrying resistance genes, antibiotics were used at the following concentrations: 100 µg $ml^{-1}$ for ampicillin, 10 µg $ml^{-1}$ for erythromycin and 5 µg $ml^{-1}$ for tetracycline. Overnight cultures were diluted in fresh medium to an optical density at 600 nm ($OD_{600}$) of 0.05 and a flask-to-medium ratio of 5:1. Cultures were grown at 37°C shaking (220 rpm). Initial growth was monitored as the $OD_{600}$ of the culture over time, whereas long-term cell survival was measured by determining the CFU $ml^{-1}$ at each time point indicated. For Figure 5 strains were first grown in CDM with Met. The cultures were filtered through a 0.22-µm filter with vacuum and washed twice with sterile phosphate-buffered saline (PBS). Bacteria were then resuspended in an equal volume of CDM with or without Met (with a flask-to-medium ratio of 2:1) and grown for another 60 min before sampled for RNA extraction.

### Construction of strains

The conditional RNase J2 (*rnjB*) and RNase III (*rnc*) mutants were generated using the pMUTIN vector [1]. With this system, conditional mutants can be obtained by integrating the vector upstream of the target gene, which falls under the control of the isopropyl IPTG-inducible promoter $P_{spac}$. For this purpose a region encompassing the first 600/900 bp of the 5′ coding region of RNase J2 and RNase III was amplified from strain RN6390 by employing the primers listed in Table S5. The amplicons were then cloned into the *Eco*RI/*Bam*HI digested pMUTIN yielding vectors pCG106 and pCG107, respectively. The vectors were transferred into the restriction-deficient strain RN4220 under IPTG induction. pMUTIN insertion into the chromosome was verified by PCR and pulsed-field gel electrophoresis (PFGE). The mutations were then transduced into *S. aureus* strain Newman. Transductants were verified by PCR and PFGE.

**RNA isolation and Northern blot analysis**

Samples from bacterial cultures were mixed with 1X Vol. RNA protection solution (Qiagen) for immediate RNA stabilization. Cells were disrupted mechanically (Bertin technologies) and total RNA was purified using mini-scale, silica-membrane based spin-columns (Qiagen).

Northern blot analysis by agarose/ formaldehyde gels of two microgramm total RNA each sample followed standard procedures. Sequence-specific probes were generated by PCR with oligonucleotides listed in Table S3 (SI) and radioactively labeled with the DNA labeling system (Amersham, GE Healthcare) and [$\alpha^{32}$P]-dCTP. For Figure 1B RNA probes were generated by T7 *in vitro* transcription with [$\alpha^{32}$P]-CTP and the DNA probe was generated by 5' end-labeling with [$\gamma^{32}$P]-ATP and polynucleotide kinase. For clean-up from unincorporated nucleotides chromatography spin-columns with Bio-Gel P-6 (Bio Rad) were used. Signals were detected with the PhosphoImager (Fujifilm FLA-7000).

For Figure 5 RNA isolation and Northern blot analysis were performed as described [2]. Briefly, bacteria were lysed in Trizol reagent (Invitrogen) with zirconia-silica beads (0.1-mm diameter) in a high-speed homogenizer (Savant Instruments, Farming- dale, NY). RNA was isolated as described by the manufacturer of Trizol. Digoxigenin (DIG)-labeled probes were generated with a DIG-labeling PCR kit following the manufacturer's instructions (Roche Biochemicals) using oligonucleotides listed in Table S3.

**Table S1 - Bacterial strains and plasmids used in this study.**

| Strain | Properties | Reference |
|---|---|---|
| ***Escherichia coli*** | | |
| DH5α | common cloning host, *lacZ* negative | |
| Top10 | Competent *E. coli* for plasmid transformation | Invitrogen |
| ***Staphylococcus aureus*** | | |
| COL | methicillin resistant isolate (1960s) | [6] |
| N315 | methicillin resistant isolate (1982) | [7] |
| Newman | methicillin-sensitive isolate (1952), NCTC 8178 | [8,9] |
| Newman, 21 | Newman *codY::tet*(M) | [10] |
| Newman, 86 | Newman $rsh_{syn}$ (nucleotides 942 to 950 deleted) | [11] |
| Newman, 106 | Newman $P_{spac}$-*rnjB*, RNase J2 mutant | this work |
| Newman, 107 | Newman $P_{spac}$-*rnc*, RNase III mutant | this work |
| RN4220 | Restriction-deficient *S. aureus* strain, r- | [12] |
| RN6390 | Laboratory strain derived from NCTC 8325 | [13] |
| ***Staphylococcus epidermidis*** | | |
| RP62A | biofilm-forming, *ica*-positive, IS*256*-positive, catheter sepsis isolate | ATCC 35984<br>[6] |

| Plasmids | Properties | Reference |
|---|---|---|
| pGEM-T Easy | linearised cloning vector system with 3' T overhang, $Amp^r$, *lacZ* | Promega |
| pGEM*met*COL | pGEM-T Easy with integration of 467 bp fragment of *met* leader RNA under T7 promoter control | this work |
| pMUTIN | Integrative vector including the IPTG-inducible promoter $P_{spac}$, $Amp^r$, $Erm^r$ | [1] |
| pCG106 | pMUTIN with integration of a 802 bp *rnjB* fragment for conditional mutagenesis | this work |
| pCG107 | pMUTIN with integration of a 600 bp *rnc* fragment for conditional mutagenesis | this work |

**Table S2 - Overview of all mutations introduced in the *met* leader RNA template.**

The numbers in the last column indicate the nucleotide position based on the *met* leader RNA sequence of *S. aureus* COL. The sequence of the oligonucleotides used for each construct are listed in Table S5.

| Construct | Oligonucleotides | Site | Mutation |
|---|---|---|---|
| **SC1** | SB_ugc_1out<br>Sa-SB_ugc_2out | **specifier box** | 113AUG → 113**UGC** |
| **SC2** | Sa-TB_del_1out<br>Sa-TB_del_2out | **T-box** | deletion of bases 360-366 |
| **SC3** | Sa-TB_acca_1out<br>Sa-TB_acca_2out | **T-box** | 360UGGU → 360**ACCA** |
| **SC4** | Sa-TB_gg361cc_1out<br>Sa-TB_gg361cc_2out | **T-box** | 361GG → 361**CC** |
| **SC5** | Sa-TB_cc365gg_1out<br>Sa-TB_cc365gg_2out | **T-box** | 365CC → 365**GG** |
| **SC6** | Sa-TB_G361C_1out<br>Sa-TB_G361C_2out | **T-box** | 361G → 361**C** |
| **SC7** | Sa-TB_G362C_1out<br>Sa-TB_G362C_2out | **T-box** | 362G → 362**C** |
| **SC8** | Sa-TB_U363A_1out<br>Sa-TB_U363A_2out | **T-box** | 363U → 363**A** |

**Table S3 - Sequences of oligonucleotides used to generate hybridization probes.**

The T7 RNA polymerase promoter sequence is underlined.

| Gene | Primer | Sequence 5' → 3' | Probe |
|---|---|---|---|
| 16S rDNA | forward | CCTTATGATTTGGGCTACACA | |
| | reverse | CCAGCTTCATATAGTCGAGTT | 130 bp |
| ***S. aureus* specific probes** | | | |
| *met* leader | forward | ATGTATTCTAAATGAGTCAGACAACC | |
| | reverse | CCGTCCTTCGTACCCGAATGA | 588 bp |
| for sense RNA probe | T7-for | TTTTCTAATACGACTCACTATAGGGAGAGGGGAGGAAAGTAAAACACACCC | |
| | reverse | CCGTCCTTCGTACCCGAATGA | 394 nt |
| for antisense RNA probe | T7-rev | TTAACTAATACGACTCACTATAGGGAGAGGGCCGTCCTTCGTACCCGAATGA | |
| | fow_nest | AGAGGAAAGTAAAACACACCCTG | 394 nt |
| *metI* | forward | GCATCCAAAACTAGGACAATCGAC | |
| | reverse | CTCTCCAATCTGAGCTTTATCTAATGC | 1006 bp |
| **DIG-labeled probes (*S. aureus* specific)** | | | |
| *brnQ-1*, permease | 02923DIG-for | GTAAAGCCCAACCAACAGGT | |
| | 02923DIG-rev | TCATCGTAGGTTTAACAGCA | 321 bp |
| *met* leader | metRIBdigfor | CTTCAAGATACCAATTACAATTTC | |
| | metRIBdigrev | TTTGTTATTCCATCGCTGA | 456 bp |
| *metI* | Nwmn_0351digfor | AAAATCCTACACGCTCAACA | |
| | Nwmn_0351digrev | GGTGTTGAAAGATAAGGTGTT | 400 bp |

**Table S4 – Nucleotide sequences of RACE primers and oligonucleotides used in tRNA template PCR.** T7 promoter sequence is underlined; additional nucleotides to the annotated tRNA sequence after the transcription reaction are highlighted in bold script.

**RACE primer**

| **Oligonucleotide** | **Sequence 5’ → 3’** |
|---|---|
| *S. aureus* specific | |
| sp1 = sp2 | CGTGCGTAAAGAATCCAGTACGCC |
| sp3 | AGACACCTCATATTGGCATCAAC |
| sp5 | AGAGGAAAGTAAAACACACCCTG |
| nested sp5 | AGTATGGGATAGCACATTACTATATCC |
| further nested sp5 | ACTGAATAAGGTTATTTCAGCGATGG |

**primer for tRNA template**

| **Oligonucleotide** | **Sequence 5’ → 3’** |
|---|---|
| Met12_tRNA_T7-F | <u>CTAATACGACTCACTATA</u>**G**CGCGGGATGGAGCAGTTCGGT |
| Met12_tRNA**cca**_R | **TGG**TTGCGGGAGGCGGATTTGAACCACC |
| Met12_tRNA**AdC**_R | **GTGG**TTGCGGGAGGCGGATTTGAACC |
| Cys_tRNA_T7-F | TTTT<u>CTAATACGACTCACTATA</u>**G**GGCGGCATAGCCAAGTGGT |
| Cys_tRNA**cca**_R | TGGAGGCGGCAACCGGATTTG |
| Cys_tRNA**AdC**_R | **G**TGGAGGCGGCAACCGGATTTG |
| Met3_tRNA_T7-F | TTAA<u>CTAATACGACTCACTATA</u>GGACCTTTAGCTCAGTTGGT |
| Met3_tRNA**cca**_R | **TGG**TGGACCTTGCAGGACTCGA |
| Met3_tRNA_R | TGGACCTTGCAGGACTCGAACC |
| Met4_tRNA_T7-F | <u>CTAATACGACTCACTATA</u>**G**GGCGGTGTAGCTCAGCTGGC |
| Met4_tRNA**cca**_R | T**G**GTGGCGGTGGAGGGGATCGAACCC |

**Table S5 - Sequences of oligonucleotides used in SDM PCR and for generation of conditional RNase mutants.** T7 promoter sequence and mutated nucleotides are underlined; position of deletion is indicated by an asterisk. The restriction sites *Eco*RI and *Bam*HI, respectively, are in italic.

**SDM PCR primers**

| **Oligonucleotide** | **Sequence 5'→ 3'** |
|---|---|
| T7-F_met-sRNA | TTAACTAATACGACTCACTATAGGGAGATCTTATAACAGTTTAATGAAACGTAAAC |
| R_met-sRNA | GAAAAAATAAAAAAAGCTTCCGTCCTTCG |
| SB_ugc_1out | GAAATGCGCCTTGGAGTGTTGATGC |
| Sa-SB_ugc_2out | AGGCGCATTTCAAACACGCTTTCAA |
| Sa-TB_del_1out | AAAGG*GCGAAACATAAGCTTTCGTCC |
| Sa-TB_del_2out | TTCGC*CCTTTATTTGTTATTCCATCGC |
| Sa-TB_acca_1out | AAAGGACCAACCGCGAAACATAAGC |
| Sa-TB_acca_2out | TTCGCGGTTGGTCCTTTATTTGTTATTCCA |
| Sa-TB_cc365gg_1out | AAAGGTGGTAGGGCGAAACATAAGC |
| Sa-TB_cc365gg_2out | TTCGCCCTACCACCTTTATTTGTTATTCCATCG |
| Sa-TB_G362C_1out | AAAGGTGCTACCGCGAAACATAAGC |
| Sa-TB_G362C_2out | TTCGCGGTAGCACCTTTATTTGTTATTCCA |
| Sa-TB_G361C_1out | AAAGGTCGTACCGCGAAACATAAGC |
| Sa-TB_G361C_2out | TTCGCGGTACGACCTTTATTTGTTATTCCA |
| Sa-TB_gg361cc_1out | AAAGGTCCTACCGCGAAACATAAGC |
| Sa-TB_gg361cc_2out | TTCGCGGTAGGACCTTTATTTGTTATTCCA |
| Sa-TB_U363A_1out | AAAGGTGGAACCGCGAAACATAAGC |
| Sa-TB_U363A_2out | TTCGCGGTTCCACCTTTATTTGTTATTCCA |

**Conditional RNase mutants**

| **RNase gene** | **Oligonucleotide** | **Sequence 5' → 3'** |
|---|---|---|
| RNase J2 | EcoSAV1275-for | GGGG*GAATTC*TAGGAGGTAAGATTTTGAG |
| | BamHSAV1275-rev | CCCC*GGATCC*TCAAGTGATCTTCCTAAA |
| RNase III | EcoSAV1233-for | GGGG*GAATTC*GCAACACATAAAGGAGAT |
| | BamHSAV1233-rev | AAAA*GGATCC*ATTATAGGTTACATCACC |

## SI References


1. Vagner V, Dervyn E, Ehrlich SD (1998) A vector for systematic gene inactivation in *Bacillus subtilis*. Microbiology 144 ( Pt 11): 3097-3104.
2. Goerke C, Campana S, Bayer MG, Doring G, Botzenhart K, et al. (2000) Direct quantitative transcript analysis of the agr regulon of *Staphylococcus aureus* during human infection in comparison to the expression profile in vitro. Infect Immun 68: 1304-1311.
3. Larkin MA, Blackshields G, Brown NP, Chenna R, McGettigan PA, et al. (2007) Clustal W and Clustal X version 2.0. Bioinformatics 23: 2947-2948.
4. Green NJ, Grundy FJ, Henkin TM (2010) The T box mechanism: tRNA as a regulatory molecule. FEBS Lett 584: 318-324.
5. Gutierrez-Preciado A, Henkin TM, Grundy FJ, Yanofsky C, Merino E (2009) Biochemical features and functional implications of the RNA-based T-box regulatory mechanism. Microbiol Mol Biol Rev 73: 36-61.
6. Gill SR, Fouts DE, Archer GL, Mongodin EF, Deboy RT, et al. (2005) Insights on evolution of virulence and resistance from the complete genome analysis of an early methicillin-resistant *Staphylococcus aureus* strain and a biofilm-producing methicillin-resistant *Staphylococcus epidermidis* strain. J Bacteriol 187: 2426-2438.
7. Kuroda M, Ohta T, Uchiyama I, Baba T, Yuzawa H, et al. (2001) Whole genome sequencing of meticillin-resistant *Staphylococcus aureus*. Lancet 357: 1225-1240.
8. Duthie ES (1952) Variation in the antigenic composition of staphylococcal coagulase. J Gen Microbiol 7: 320-326.
9. Baba T, Bae T, Schneewind O, Takeuchi F, Hiramatsu K (2008) Genome sequence of *Staphylococcus aureus* strain Newman and comparative analysis of staphylococcal genomes: polymorphism and evolution of two major pathogenicity islands. J Bacteriol 190: 300-310.
10. Pohl K, Francois P, Stenz L, Schlink F, Geiger T, et al. (2009) CodY in *Staphylococcus aureus*: a regulatory link between metabolism and virulence gene expression. J Bacteriol 191: 2953-2963.
11. Geiger T, Goerke C, Fritz M, Schafer T, Ohlsen K, et al. (2010) Role of the (p)ppGpp synthase RSH, a RelA/SpoT homolog, in stringent response and virulence of *Staphylococcus aureus*. Infect Immun 78: 1873-1883.
12. Kreiswirth BN, Lofdahl S, Betley MJ, O'Reilly M, Schlievert PM, et al. (1983) The toxic shock syndrome exotoxin structural gene is not detectably transmitted by a prophage. Nature 305: 709-712.
13. Peng HL, Novick RP, Kreiswirth B, Kornblum J, Schlievert P (1988) Cloning, characterization, and sequencing of an accessory gene regulator (agr) in *Staphylococcus aureus*. J Bacteriol 170: 4365-4372.
